# Dimensional Accuracy and Clinical Fitness of Customized 3D-Printed Hydroxyapatite Bone Block Grafts in Alveolar Ridge Augmentation

**DOI:** 10.1055/s-0045-1812864

**Published:** 2025-11-17

**Authors:** Pichaya Mekcha, Faungchat Thammarakcharoen, Jintamai Suwanprateeb, Lucy Di Silvio, Borvornwut Buranawat

**Affiliations:** 1 Division of Periodontics and Implant Dentistry, Faculty of Dentistry, Thammasat University, Khlong Luang, Pathum Thani, Thailand; 2 Biofunctional Materials and Devices Research Group, National Metal and Materials Technology Center, National Science and Technology Development Agency, Pathum Thani, Thailand; 3 Thammasat University Center of Excellence in Computational Mechanics and Medical Engineering, Thammasat University, Pathum Thani, Thailand; 4 Center for Oral, Clinical and Translational Sciences, Faculty of Dentistry, Oral & Craniofacial Sciences, King's College London, London, United Kingdom; 5 Department of Implantology, Faculty of Dentistry, Thammasat University, Pathum Thani, Thailand

**Keywords:** hydroxyapatite, 3D printing, alveolar ridge augmentation, dimensional accuracy, binder jetting technique, bone block grafts

## Abstract

**Objective:**

This article evaluates the feasibility of manufacturing customized three-dimensional (3D)-printed hydroxyapatite (3DHA) bone block grafts using binder jetting for alveolar ridge augmentation prior to implant placement.

**Materials and Methods:**

Cone-beam computed tomography (CBCT) scans of the alveolar ridge from eight patients with horizontal ridge defects were converted to STL files to generate 3D models. These models were used to design and fabricate patient-specific 3DHA bone block grafts using binder jetting. The 3D-printed grafts underwent micro-CT (μCT) scanning, generating μSTL files, which were compared with the original computer-aided design models using inspection software. Dimensional accuracy in three axes ( *x* , *y* , *z* ) and discrepancies between the 3DHA grafts and ridge defect geometries were assessed. The 3DHA grafts were clinically evaluated for effectiveness in augmenting alveolar ridge defects.

**Results:**

Percentage relative differences between the original STL files and those from μCT were –2.72, –7.24, and –3.26% for width, length, and height, respectively. Root mean square deviation was 0.19 ± 0.04 mm overall, –0.22 ± 0.25 mm for the margin area, –0.08 ± 0.18 mm for the inner area, and 0.03 ± 0.11 mm for the contour. Mean gap distances at the defect site were 0.19 ± 0.22 mm (marginal) and 0.37 ± 0.12 mm (internal), which are within clinical acceptable ranges. Clinical application showed no need for intraoperative graft adjustment. At 6 months postaugmentation, bone volume increased from 164.4 ± 37.87 mm ^3^ (initial μCT) to 169.2 ± 39.38 mm ^3^ (follow-up CBCT), though this change was not statistically significant ( *p* = 0.0538). The mean dimensional change was 0.30 ± 0.14 mm.

**Conclusion:**

In this pilot study, 3DHA bone block grafts demonstrated acceptable dimensional accuracy and were successfully used for alveolar ridge augmentation, indicating their potential for patient-specific applications.

## Introduction

Synthetic graft has emerged as an alternative to traditional autogenous bone harvesting in dental and maxillofacial procedures. While autogenous bone block grafts remain the gold standard for bone augmentation, they are associated with several drawbacks, including patient morbidity, technical complexity, and time-consuming procedures. 1 2 These limitations have driven the search for alternative bone graft materials that can provide comparable outcomes with reduced patient burden.

Xenografts, typically derived from bovine or porcine sources, are natural in origin. 3 4 They maintain much of the original bone's structure and composition, including the mineral content and collagen matrix. This similarity to human bone often allows for excellent biocompatibility and osteoconductive properties. 5 The natural origin of xenografts may contribute to their steady and predictable bone density increase over time. 6 Hydroxyapatite (HA), a synthetic composite material, has gained prominence in the field of bone regeneration. 7 Its chemical structure closely resembles that of natural alveolar bone, making it an excellent candidate for bone substitution. HA was developed to replicate the inorganic components of bone tissue in a controllable, manufactured form. 8 This synthetic nature allows for precise control over its composition, porosity, and degradation rate, optimizing bone regeneration. 8

HA belongs to the family of calcium phosphate biomaterials, widely used for the treatment of bone defects due to their osteoconductive properties and biocompatibility. Its advantages include strong bioaffinity and osteoconductive properties, which create a favorable environment for osteoprogenitor cells to proliferate and differentiate into bone-forming cells. 9 However, properties such as pore size, configuration, and sintering temperature significantly influence HA's performance. 10 When designed with an optimal pore size and shape, HA can provide a three-dimensional (3D) structure for bone ingrowth while maintaining mechanical durability. 10 However, HA still has certain limitations. Traditional sintering-based manufacturing techniques can lead to slow resorption rates. Despite these challenges, ongoing research continues to improve HA's properties and performance making it a valuable tool in bone augmentation, repair, and regeneration procedures. 11

Recently, 3D printing technology has revolutionized the field of bone grafting by offering a personalized and efficient approach. Advanced imaging techniques, such as cone-beam computed tomography (CBCT), allow clinicians to generate a detailed 3D model of the patient's alveolar bone and the specific area requiring grafting. This digital model is then used to design and fabricate a customized bone graft using biocompatible materials such as HA or polylactic acid, ensuring a precise fit tailored to the patient's anatomical needs. 12

Previously, we have developed a 3D-printed HA (3DHA), as a potential alternative to autogenous bone grafts. The 3DHA bone graft is fabricated using a binder jetting technique with low-temperature phosphorization, designed to serve as a bone substitute for alveolar ridge augmentation before implant placement. 13 14 A key advantage of this 3DHA lies in the integration of 3D printing technology, which enables the fabrication of grafts in precise shapes and sizes tailored to alveolar defects. In addition, its resorbability supports tissue regeneration and promotes new tissue ingrowth. 15 16 17

While the use of 3DHA in bone regeneration has been reported, studies quantitatively integrating high-resolution dimensional accuracy analysis with initial clinical outcomes are still limited. In clinical applications, the success of 3D-printed bone block grafts depends on various factors including their fit to the recipient site defect. Several methods are available for analyzing scaffold structure. 18 One of the most effective techniques is micro-CT (µCT), which allows detailed visualization of bone architecture and provides a 3D assessment of scaffold morphology. 19 µCT offers high-resolution imaging and is widely accessible for evaluating bone graft integrity and internal structure. 20 To assess the accuracy of 3D-printed grafts, 3D analysis software was used to compare the printed bone graft scan with the original virtual design through superimposition techniques. 21 This method enables precise evaluation of fabrication accuracy, particularly for materials with irregular or complex geometric shapes. 22 Recently, 3D assessment techniques have been introduced in prosthetic dentistry for applications such as evaluating the fit of dental crowns and metal frameworks for removable partial dentures. 23 24 However, limited data exist on the accuracy of this tool in prosthetic dentistry, highlighting the need for further research in this area.

This pilot study aims to evaluate the dimensional accuracy of 3DHA bone block grafts, manufactured using the binder jetting technique, by assessing their internal fit to the target defect and comparing the 3D deviation between the printed grafts and their virtual designs. Clinical application was preliminary conducted in eight patients, where the optimized 3DHA grafts were assessed for their efficacy in augmenting alveolar ridge defects. This study uniquely advances the field by demonstrating quantitative dimensional accuracy alongside initial clinical outcomes, establishing both the technical precision and translational feasibility of this approach.

## Materials and Methods

### Study Workflow

This study utilized a four-step approach to evaluate dimensional accuracy and fit (gap between the 3DHA graft and the ridge defect), including: (1) designing the 3DHA graft based on individual patient CBCT scans; (2) scanning the fabricated 3DHA graft using μCT; (3) assessing the dimensional accuracy of the printed graft; and (4) analyzing the gap between the 3DHA graft and the intended ridge defect ( Fig. 1A ).

**Fig. 1:**
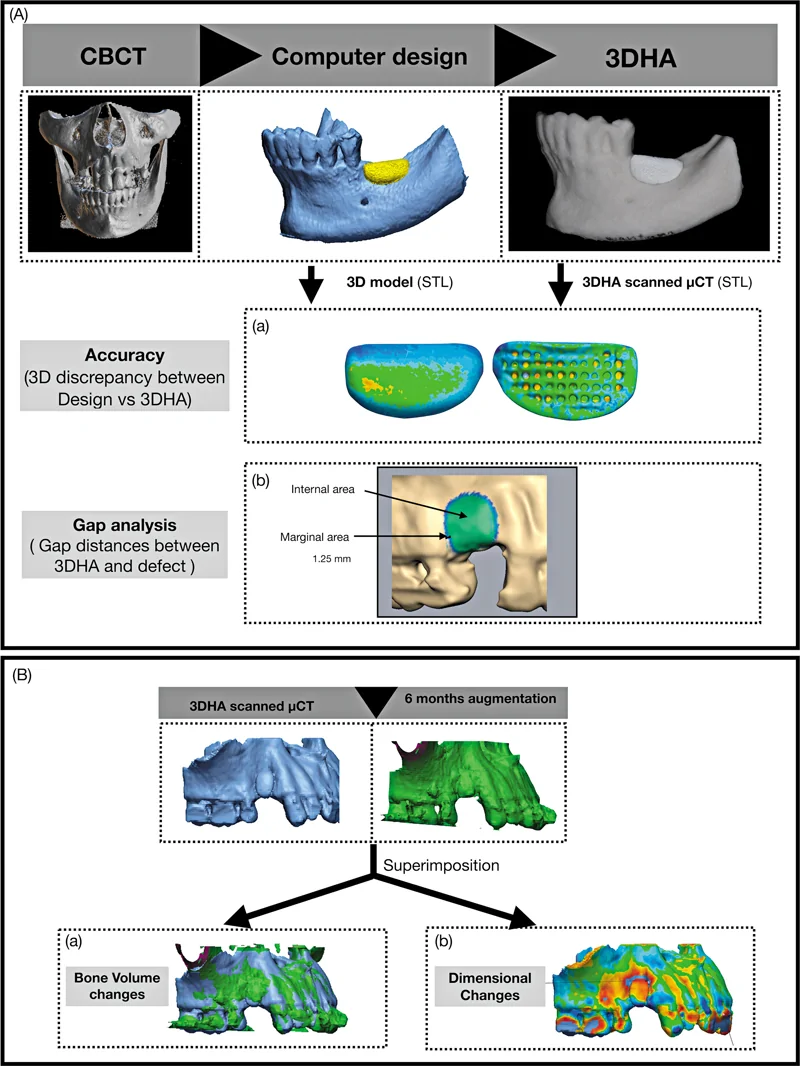
( **A** ) Study workflow to analyze printing accuracy, fitness, and gap of three-dimensional (3D)-printed hydroxyapatite (3DHA) grafts: (a) 3D discrepancy between the virtual 3DHA design and the microcomputed tomography (µCT)-scanned 3DHA is illustrated using color maps to show deviation patterns, (b) STL files of the scanned 3DHA obtained from μCT were aligned with the STL files of patients' defects. The marginal gap was measured 1.25 mm from the marginal edge (blue area), while the internal gap was measured in the remaining internal graft region, excluding the 1.25-mm margin (green area). ( **B** ) Assessment of bone volume and dimensional changes at 6 months postaugmentation through superimposition of STL files from μCT-scanned 3DHA and 6 months cone-beam computed tomography (CBCT) scans: (a) bone volume changes, (b) dimensional changes.

Clinical data were collected to evaluate changes in bone volume and dimensions. The STL data sets from μCT (initial bone engraftment) and CBCT (6 months postaugmentation) were analyzed to quantify these changes ( Fig. 1B ). No calibration or cross-validation was performed to account for the differences between these imaging modalities in voxel size, resolution, and reconstruction algorithms.

#### 3DHA Bone Block Graft Design

CT scans of the patients were obtained with ethical approval from the Human Research Ethics Committee of Thammasat University (Science) (COA no. 039/2562). The eight alveolar ridge defects were located in the anterior maxilla (3 cases), premolar maxilla (4 cases), and posterior mandible (1 case). Digital Imaging and Communications in Medicine (DICOM) files from the scans were imported into a dental implant planning software (DentiPlan, NECTEC, Thailand) and then converted into STL files. These STL files were further processed using 3D design software (Geomagic Freeform, 3D Systems, United States) and a professional haptic device (The Touch Haptic Device, 3D Systems) to create customized 3D bone block grafts. The 3DHA grafts were manufactured layer-by-layer by a binder jetted 3D printer (Projet160, 3D Systems) operating at a resolution of 300 × 450 dpi and a layer thickness of 0.1 mm. Calcium sulfate-based powder (Visijet PXL Core, 3D Systems) and a water-based binder (Visijet PXL Clear, 3D Systems) were employed as raw materials. During printing, the binder selectively adhered the powder particles at designated regions to form the structure, which solidified through evaporation, eliminating the need for additional curing. No postprocessing was performed. After the grafts were printed, they were soaked in 1 M disodium hydrogen phosphate solution (Sigma Aldrich, United States) at 100°C for 48 hours, resulting in conversion to HA structure. This technique preserved the original dimensions of the grafts and avoided the shrinkage typically associated with sintering. The 3DHA possesses a porosity of 82.09 ± 3.00% and demonstrate a compressive strength of 7.32 ± 0.54 N. Finally, all 3DHA grafts were dried and sterilized using ethylene oxide gas. 17

#### Microcomputed Tomography

The 3DHA were scanned using μCT scanner (SkyScan 1275, Bruker, Kontich, Belgium) to assess their 3D morphology and architecture of 3DHA. Each 3DHA was securely mounted on a holder table for scanning. The scanning procedure was performed using an X-ray source potential of 70 kV, a current of 129 μA, 8W, and a voxel size of 6 μm. X-ray transmission through the 3DHA samples was acquired over a full 360-degree rotation, with an exposure time of approximately 30 minutes per sample.

All projection image data were reconstructed using NRecon software (NRecon V.2, Skyscan, Bruker) to generate 3D images, which were visualized using CTvox software (CTvox, Skyscan, Bruker). The volume of interest of the 3DHA was then analyzed using CTAn software (CTAn, Skyscan, Bruker) and converted into STL files for further evaluation ( Fig. 2 ).

#### Dimensional Accuracy Assessment

The geometric dimensions (width, length, height) of the 3DHA bone block graft virtual design and μCT-scanned models were measured using a dimensional bounding box, aligned to the global coordinate axis ( Supplementary Fig. S1 , available in the online version only).

Measurements were recorded in millimeter (mm) using Geomagic FreeForm (3D Systems). The relative percentage differences between the virtual design and μCT-scanned models were calculated using the following formula:



The virtual 3DHA, designed using the Geomagic FreeForm software (3D Systems), was imported into the analysis program (Geomagic Control X, 3D Systems) as reference data. The scanned 3DHA, obtained by μCT, was then superimposed on the reference data using initial alignment followed by best-fit alignment. The accuracy of the 3DHA was analyzed by calculating the root mean square (RMS) of the total graft area (overall) as well as dividing into three sections: marginal, contour, and internal area each consisting of 40 points (point-to-surface). A 3D analysis was performed to assess the deviation between the scanned 3DHA and the reference model.

The measurement data were evaluated using the intraclass correlation coefficient at 95% confidence interval to determine the level of accuracy of the printed 3DHA graft within the same tested groups. The standard error of repeated measurement was less than 0.002 mm. For the 3D analysis, the differences between the superimposed data were analyzed using the 3D compare function in the inspection software, which generated color surface maps. A maximum critical value of ± 0.10 mm and maximum nominal values of ± 0.01 mm were set for color spectrum.

All points within the scanned graft of the reference data and all points within the scanned 3DHA were calculated to determine the RMS values as follows:



*X*_1_ in this equation is the data point of reference (virtual design) *i* , *X*_2_ is the data point of study group *i* (μCT-scanned models), and *n* is the number of all measurement points.

To better illustrate accuracy differences, a color difference map was generated using a ± 0.1 mm range (divided into 20 color segments) within the 3D inspection software. In the color map, the red zone (0.01–0.1 mm) represents a positive error, indicating that the scanned 3DHA data was larger than the reference or positive error in the printing process. Conversely, the blue zone (−0.1 to −0.01 mm) indicates a negative error, meaning the scanned 3DHA was smaller than the reference or there was shrinkage in the printing process. The green zone (±0.1 mm) reflects exceptional accuracy with meticulous precision ( Fig. 1Aa ).

#### Gap Analysis

The STL files of the scanned 3DHA obtained from μCT were aligned with the STL files of patients' defects. The mean gap distances between the internal area of the scanned 3DHA and the defect (surface-to-surface area) were assessed using Geomagic software. The internal fit evaluation was performed in two areas consisting of the marginal and internal area. The marginal gap was measured at a distance of 1.25 mm from the marginal edge (blue area). The internal gap was measured at the internal graft area, excluding the 1.25 mm margin (green area), as shown in Fig. 1Ab .

#### Bone Volume and Dimensional Changes

The CBCT scan (DentiiScan 2.0, NSTDA, Thailand) was taken at 6 months postaugmentation (voxel size 0.2 mm, field of view [FOV] [diameter × height] 6 × 6 cm ^2^ , focal spot 0.5 mm, 70 kVp). The DICOM data set was converted to an STL file data set. For the baseline data, the initial graft from μCT was reconstructed using CTAn software (CTAn, Skyscan, Bruker) and converted into STL format, then merged with the preoperative bone defect model. The initial μCT model with the defect was superimposed onto 6-month CBCT model. To determine the bone volume changes, the 6-month CBCT volume was subtracted from the initial μCT volume and volume changes were calculated in mm ^3^ using Geomagic FreeForm software ( Fig. 1Ba ).

Dimensional changes were analyzed by measuring the 3D horizontal ridge alterations between the baseline μCT and the 6-month postaugmentation CBCT data in mm using Geomagic Control X software ( Fig. 1Bb ).

#### Clinical Relevance

Eight patients who required dental implant therapy with horizontal bone defect were enrolled in this study between June 2019 and December 2020 at the Faculty of Dentistry, Thammasat University Hospital. The clinical protocol was registered in the Thai Clinical Trials Registry (Study ID: TCTR20190704004) and approved by the Human Research Ethics Committee of Thammasat University (Science) (COA no. 039/2562). Written informed consent, outlining the details and purpose of the study, was obtained from all patients.

### Statistical Analysis

Statistical analysis was performed using GraphPad Prism 9.0.0 (GraphPad, California, United States). Descriptive statistics were presented as mean and standard deviation (SD). The mean of geometric data for the 3DHA virtual design and scanned µCT was presented as width, length, and height in millimeters (mm). The relative differences were expressed as percentages. 3D comparison between the µCT scan of 3DHA and the virtual design was presented as mean RMS data (mm). Reliability was assessed using the intraclass correlation coefficient. The Shapiro–Wilk test was applied to determine the normal distribution of the RMS (mm) data for 3D analysis of each surface. A repeated-measures one-way analysis of variance (ANOVA) followed by multiple comparisons test was used to evaluate the RMS data for accuracy assessment of all-eight-graft at: (1) the overall area, (2) marginal area, (3) inner area, and (4) contour areas. A paired *t* -test was used to analyze bone volume changes between the initial μCT and 6-month postaugmentation CBCT. A *p* -value of < 0.05 was considered statistically significant.

## Results

### Geometric Data Analysis of 3DHA

The STL files illustrated the 3DHA virtual design ( Supplementary Fig S2A , available in the online version only) and scanned µCT model ( Supplementary Fig S2B , available in the online version only) for each defect (defects 1 to 8). The geometric measurements, including width, length, and height, are presented as mean ± SD in Table 1 . The data revealed that µCT values were lower than the corresponding 3DHA-designed dimensions across all defects. The relative differences were minimal for width (–2.72%) and height (–3.26%), while a greater difference was observed in length (–7.24%).

**Table 1:** Geometric data of 3DHA virtual design and scanned µCT, presented as width, length, and height in millimeters (mm) along with the relative differences in percentages

Defect	Width (mm)			Length (mm)			Height (mm)		
	Design	µCT	Relative difference	Design	µCT	Relative difference	Design	µCT	Relative difference
1	6.89	6.73	–2.32%	16.82	16.28	–3.21%	9.06	8.89	–1.88%
2	6.74	6.56	–2.67%	7.73	7.01	–9.31%	10.94	10.61	–3.02%
3	5.73	5.62	–1.92%	12.06	11.01	–8.71%	8.94	8.61	–3.69%
4	5.45	5.13	–5.87%	8.83	8.2	–7.13%	11.18	11	–1.61%
5	8.98	8.78	–2.23%	7.92	7.13	–9.97%	10.59	10.22	–3.49%
6	8.71	8.43	–3.21%	10.24	9.25	–9.67%	11.62	11.04	–4.99%
7	7.53	7.45	–1.06%	10.46	9.86	–5.74%	9.34	8.9	–4.71%
8	7.81	7.62	–2.43%	12.93	12.39	–4.18%	10.87	10.58	–2.67%
Mean ± SD	7.23 ± 1.28	7.04 ± 1.28	–2.72%	10.87 ± 3.03	10.14 ± 3.09	–7.24%	10.32 ± 1.04	9.98 ± 1.02	–3.26%

Abbreviations: 3DHA, three-dimensional-printed hydroxyapatite; µCT, microcomputed tomography; SD, standard deviation.

### 3D Comparison Between the Virtual 3DHA Design and the µCT Scanned 3DHA

The 3D color maps illustrated the deviation pattern between the virtual 3DHA design and the µCT-scanned 3DHA. The deviation pattern exhibited a heterogeneous distribution. Shrinkage (negative RMS value) of 3DHA was observed in the marginal area. The accuracy (precision by RMS value) was highest in the inner and outer areas.

The overall accuracy of 3DHA, measured by the RMS value (mean ± SD), mm, for *n* = 8, was 0.19 ± 0.04 mm. The mean distance value for the 40-point measurement in the marginal area was –0.22 ± 0.25 mm, indicating shrinkage, as represented by light blue to dark blue in the color map. In the internal area, the mean distance value was –0.08 ± 0.18 mm, with green to light blue regions reflecting precise 3DHA printing. The contour area had a mean distance value of 0.03 ± 0.11 mm, also depicted in green to light blue, further confirming printing accuracy ( Fig. 3 ), see also Supplementary Table S1 , available in the online version only. The intraclass correlation coefficient for these measurements ranged from 0.87 to 0.99, indicating an almost perfect agreement.

**Fig. 2:**
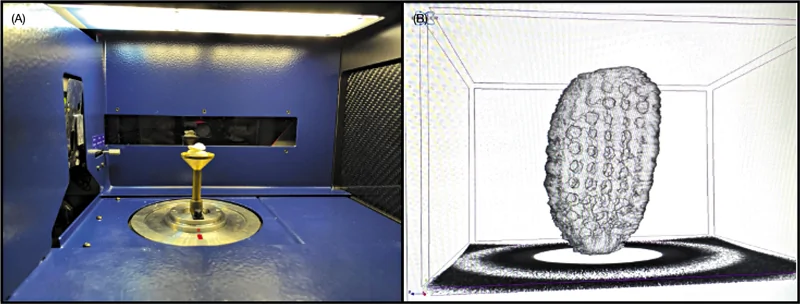
Three-dimensional (3D)-printed hydroxyapatite (3DHA) grafts were scanned using microcomputed tomography ( **A** ) and subsequently analyzed with CTAn software (CTAn, Skyscan) to generate STL files for further evaluation ( **B** ).

**Fig. 3:**
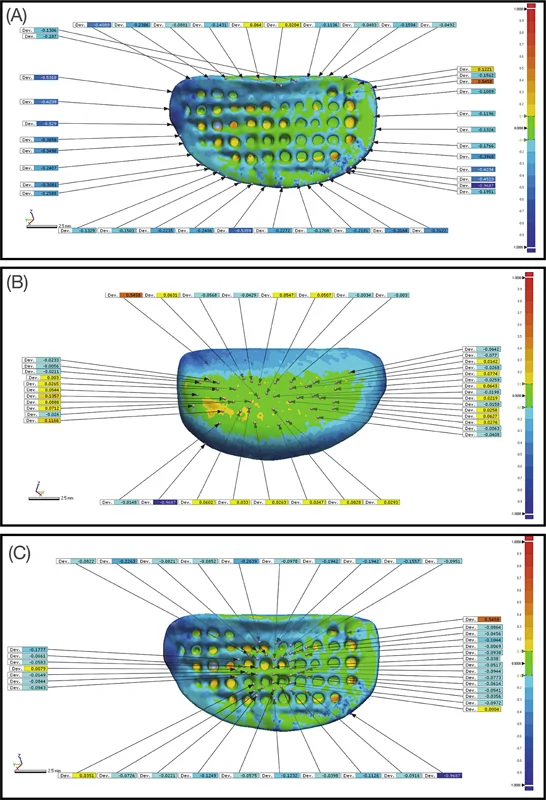
Measurement of 40 points on the marginal ( **A** ), internal ( **B** ), and contour ( **C** ) areas of three-dimensional (3D)-printed hydroxyapatite (3DHA). In the color map, red indicates a positive deviation, showing that the scanned 3DHA was larger than the reference model. Blue (−0.1 to −0.01 mm) indicates a negative deviation, where the scanned 3DHA was smaller than the reference. Green (±0.1 mm) represents areas of high-dimensional accuracy, reflecting the precision of the printing process.

The Shapiro–Wilk test was used to assess the normality of all data. The data distribution met the assumption of log-normality for all measured values ( *α* = 0.05). A repeated-measures ANOVA revealed a statistically significant difference in RMS values for the overall, margin, inner, and contour regions of the graft. The multiple comparisons test also showed that the overall RMS differed significantly ( *p* < 0.0001) from the margin, inner, and contour regions of the graft, as presented in Supplementary Table S2 , available in the online version only.

### Gap Distances Between 3DHA and Defect Site

The mean gap distance between the 3DHA and defect site was 0.14 ± 0.39 mm. Minimal or no gap at the marginal area of the graft (0.19 ± 0.22 mm), while the gap distances between the 3DHA and defect at the internal area (0.37 ± 0.12 mm), Supplementary Table S3 , available in the online version only. The gap at the marginal interest area and cross-sectional plane at the middle part of 3DHA are illustrated in Supplementary Figs S3 and S4 , respectively (available in the online version only).

In conclusion, the fit between the 3DHA graft and the defect site was shown to be accurate at the marginal area, but the differences were not statistically significant ( *p* = 0.1094). According to a previous study, 17 the marginal gaps error between 200 and 300 µm (0.2–0.3 mm) was clinically acceptable.

### Clinical Results

The 3DHA bone block graft, when placed into the patient's defect, showed a good fit with the recipient side wall. The 3DHA did not require any trimming or adjustment, demonstrating its suitability for the defect site, Supplementary Fig S5 , available in the online version only.

### Bone Volume and Dimensional Changes

Bone volume changes at 6 months postaugmentation showed an increase from the initial graft volume measured by µCT (164.4 ± 37.87 mm ^3^ ) to the volume measured by CBCT at 6 months postaugmentation (169.2 ± 39.38 mm ^3^ ). This change approached, but did not reach, statistical significance ( *p* = 0.0538). The mean dimensional change was 0.30 ± 0.14 mm, Table 2 .

**Table 2:** Bone volume and dimensional changes between initial graft and 6 months

Defect	Initial graft volume by µCT (mm ^3^ )	6 mo Postaugmentation by CBCT (mm ^3^ )	Dimensional changes (mm; mean ± SD)
1	201.2705	207.22	0.33 ± 0.04
2	117.9857	119.89	0.16 ± 0.07
3	161.0382	167.29	0.38 ± 0.07
4	143.555	150.11	0.41 ± 0.16
5	122.0555	124.89	0.16 ± 0.04
6	201.8797	209.81	0.54 ± 0.16
7	150.8579	152.6	0.13 ± 0.05
8	216.3364	221.4	0.30 ± 0.04
Mean ± SD	164.4 ± 37.87	169.2 ± 39.38	0.30 ± 0.14

Abbreviations: µCT, microcomputed tomography; CBCT, cone-beam computed tomography; SD, standard deviation.

## Discussion

This study mainly focuses on the 3D printing of HA for clinical applications. The use of bioceramic materials, particularly HA, as bone grafts can vary in mechanical properties due to factors such as pore size, graft size, interconnecting layers, and the manufacturing process. The traditional method for producing HA involves sintering at high temperatures, which results in lower bioactivity, slower resorption rates, reduced specific surface area, and lower porosity. In contrast, HA produced using low-temperature techniques exhibits higher bioactivity, faster bioresorption, greater specific surface area, and increased porosity. 25 26 27 Furthermore, 3DHA features microporosity that improves water permeability and enhances surface area topography. This nanohydroxyapatite scaffold gradually degrades or is resorbed over time, creating space for new bone formation. 13 26 Due to the few studies of 3D bone printing accuracy, this study was focused on the accuracy evaluation of 3DHA bone graft after printing, compared with the original design, and some applications to the clinic.

The accuracy of 3D printing refers to how closely the dimensions of a printed scaffold match its original model, as represented by nominal values. In this study, the first method to evaluate dimensional accuracy involves geometric measurements of width, length, and height from the STL data of 3DHA after printing, compared with the original computed design, due to the irregular shape of 3DHA. Therefore, dimensional accuracy is crucial for assessing a machine's reliability in producing objects that meet expected specifications. This study investigates how the 3D printing process affects the dimensional accuracy of scaffolds manufactured using this technology. The original design was created using computer software, and the scaffolds were analyzed after the 3D printing process; subsequently, they were scanned using µCT imaging. The results indicate that the scaffolds experienced a shrinkage of approximately 2 to 7% compared with the original design after printing. The shrinkage of the grafts occurred in all dimensions (width, length, and height), as shown by the negative values of the percentage of relative difference: –2.72% for width, –7.24% for length, and –3.26% for height. The greatest shrinkage was observed in length, likely because the largest side of the graft is more susceptible to printing errors.

The other method to evaluate the dimensional accuracy is 3D comparison from Geomagic Control X software with superimposition algorithms in the automated best-fit alignment to present the 3D differences between the original graft design and the printed graft. The advantages of this program were reliable, reproducible, and easy to present in color maps to analyze the interested area. The mean values of difference shown in RMS in the interested area of overall, margin, inner, and contour area were 0.19 ± 0.04, –0.22 ± 0.25, –0.08 ± 0.18, and 0.03 ± 0.11 mm, respectively. RMS of overall differed significantly from margin ( *p* < 0.0001), inner ( *p* < 0.0001), and contour ( *p* < 0.0001). The mean of RMS was a negative value, which means shrinkage of the graft after printing at the margin and the inner of the graft. But these lower values are clinically acceptable. 28

The evaluation of the printed grafts positioned at the defect site revealed that the mean gap measurement in the marginal and internal areas was 0.19 ± 0.22 mm and 0.37 ± 0.12 mm, respectively. The comparison of the fitness between the 3DHA graft and the defect site indicated that the gap distances in the marginal areas were not statistically significant ( *p* -value = 0.109). Other studies on the gap error of printed prostheses have suggested that the marginal gaps of 200 to 300 µm (0.2–0.3 mm) are considered clinically acceptable. The accuracy of 3DHA may vary based on the initial resolution of the 3D image from patients' CBCT and the method used for manufacturing. It is possible to refine the bone defect model according to the patient's bone surface. Customized 3D bone printing from these models allows for the precise placement of bone defects and provides good marginal adaptation.

This dimensional analysis showed superior adaptation at the marginal regions compared with internal zones, with significantly reduced gap measurements at the periphery. Enhanced marginal integrity is crucial for graft stabilization and optimal integration at the recipient site without the need for using fixation pins. In all cases, the scaffolds exhibited precise conformity to defect topography, eliminating the necessity for intraoperative adjustments or modifications. Conversely, traditional autogenous or allograft bone blocks require substantial intraoperative manipulation and reshaping to achieve proper defect adaptation, directly increasing surgical duration and consequent patient morbidity. 1 29

Large-volume alveolar ridge augmentation, especially vertical ridge augmentation, is a challenging surgical procedure due to the lack of support from adjacent bony walls. 12 In this study, the 3D-printed bone block graft was used exclusively for horizontal ridge augmentation, with 3DHA precisely adapted to the bony walls of the defects. The 3D printing technique was applied to areas involving no more than three teeth. Consequently, the use of 3D-printed bioresorbable scaffolds for bone augmentation in clinical settings has been limited.

This study demonstrated changes in bone volume and dimensional changes using both µCT and CBCT imaging techniques. Both methods provided a 3D morphology of the bone graft but with different resolution. One study found that CBCT can be used for a clinically reliable assessment of alveolar bone quality, achieving accuracy and reliability levels comparable to those of µCT. 30 31 Additionally, another study concluded that CBCT provides comparable accuracy to high-resolution µCT when assessing trabecular structures in the mandible. 32 33 In a comparison of the accuracy of 3D-printed models of the mandible derived from CBCT systems with different FOVs, it was discovered that smaller voxels provided the highest precision and greater accuracy in reconstructing maxillofacial landmarks. 34 This study indicated that while CBCT is a feasible tool for such studies, the differences in resolution between CBCT and µCT still limit the interpretation of results.

The 3DHA in this study demonstrated greater resorption than sintered HA, which is typically characterized by limited resorption capacity. 14 26 The additional xenograft of a 1-mm layer over the 3DHA was used to protect against bone resorption. The results showed differences in graft volume between the initial measurement from µCT (164.4 ± 37.87 mm ^3^ ) and a follow-up measurement at 6 months postaugmentation using CBCT (169.2 ± 39.38 mm ^3^ ). Furthermore, the factors influencing volumetric changes are likely not derived from the original bone block graft itself but may be influenced by the overlay of the xenograft and platelet-rich fibrin (PRF) membrane. These findings demonstrate the feasibility of gaining bone volume after augmentation.

Another study on 3D-printed titanium mesh for treating alveolar ridge defects found that the size of the bone defect did not affect the printing accuracy. 35 This was attributed to the rigid properties of the mesh, as there was minimal variation in defect sizes among the groups, which involved the loss of one to five teeth. While this study used bioceramic materials or nanohydroxyapatite as bone graft substitutes, the mechanical properties of these materials can influence dimensional changes over time.

To address this situation, 3DHA were designed with an outer cortex that has a thickness of 2 mm, enhancing the mechanical performance of the printed parts. These constructions feature a macroporous structure that mimics the natural porosity of alveolar bone, allowing for improved blood supply and nutrient transport into the bone graft. The clinical dimensional stability of the 3DHA bone block graft after 6 months shows that the mean changes in dimensional contour were 0.30 ± 0.14 mm. These minimal contour changes in the ridge defect following the bone graft may be attributed to the addition of a 1-mm layer of xenograft, which was incorporated to slow down the resorption of the graft. 29

To enhance the success of bone augmentation, a collagen membrane (RTM collagen membrane, Cytoplast) was used to cover the contour of 3DHA due to its long resorption period of 26 to 38 weeks and lower risk of exposure compared with nonresorbable membranes. 36 37 Additionally, PRF is derived from a patient's own blood and contains a high concentration of platelets and leukocyte cytokines. 38 This concentrate is rich in growth factors, including platelet-derived growth factors, transforming growth factor, vascular endothelial growth factor, insulin-like growth factor, and epidermal growth factor. 39 PRF has been proposed for various applications in dentistry, such as alveolar ridge reconstruction, sinus lift procedures, and extraction socket preservation. 40 41 However, the effectiveness of PRF for bone regeneration remains uncertain. 42 43 44 The clinical protocol in this study involved the use of PRF due to its positive effects on soft tissue healing, as in the previous study. 17

This study has several limitations that warrant consideration. First, the small sample size of eight patients and the relatively short follow-up period of 6 months reduce the statistical power and limit the generalizability of the findings concerning the efficacy of the 3DHA bone block graft. Second, the simultaneous utilization of xenografts and PRF membranes alongside the 3DHA graft introduces potential confounding factors, making it challenging to isolate the specific effects of the 3DHA material on bone volume and dimensional changes. Third, methodological variability arises from the use of different imaging modalities to assess graft dimensions: baseline measurements were obtained via µCT, while follow-up evaluations employed CBCT. The inherent differences in voxel size, resolution, and image reconstruction between µCT and CBCT could introduce measurement bias, affecting the accuracy of longitudinal comparisons. Finally, the absence of a control group encompassing other grafting materials, such as autogenous and allogenic grafts, restricts direct comparative analysis and limits the interpretation of the 3DHA graft's performance.

## Conclusion

This pilot study demonstrated the feasibility of producing customized binder-jetted HA block grafts with submillimetric accuracy, which could be placed clinically without intraoperative modification. The workflow highlights the innovative application of binder jetting in graft fabrication, offering a high degree of customization and accuracy. Although volumetric changes observed over 6 months were small and not statistically significant, the results indicate stability during the early healing phase. To establish clinical reliability and confirm the long-term advantages of this approach, larger controlled studies with standardized volumetric and histological outcomes are warranted.
