# Supplementary material for: Dimensional Accuracy and Clinical Fitness of Customized 3D-Printed Hydroxyapatite Bone Block Grafts in Alveolar Ridge Augmentation
Source: Eur J Dent. 2025 Nov 17;20(4):1233–43. doi: 10.1055/s-0045-1812864 (PMC13623481; doi:10.1055/s-0045-1812864)
Supplement: Supplementary file 1 [file 10-1055-s-0045-1812864-s2584469.pdf]

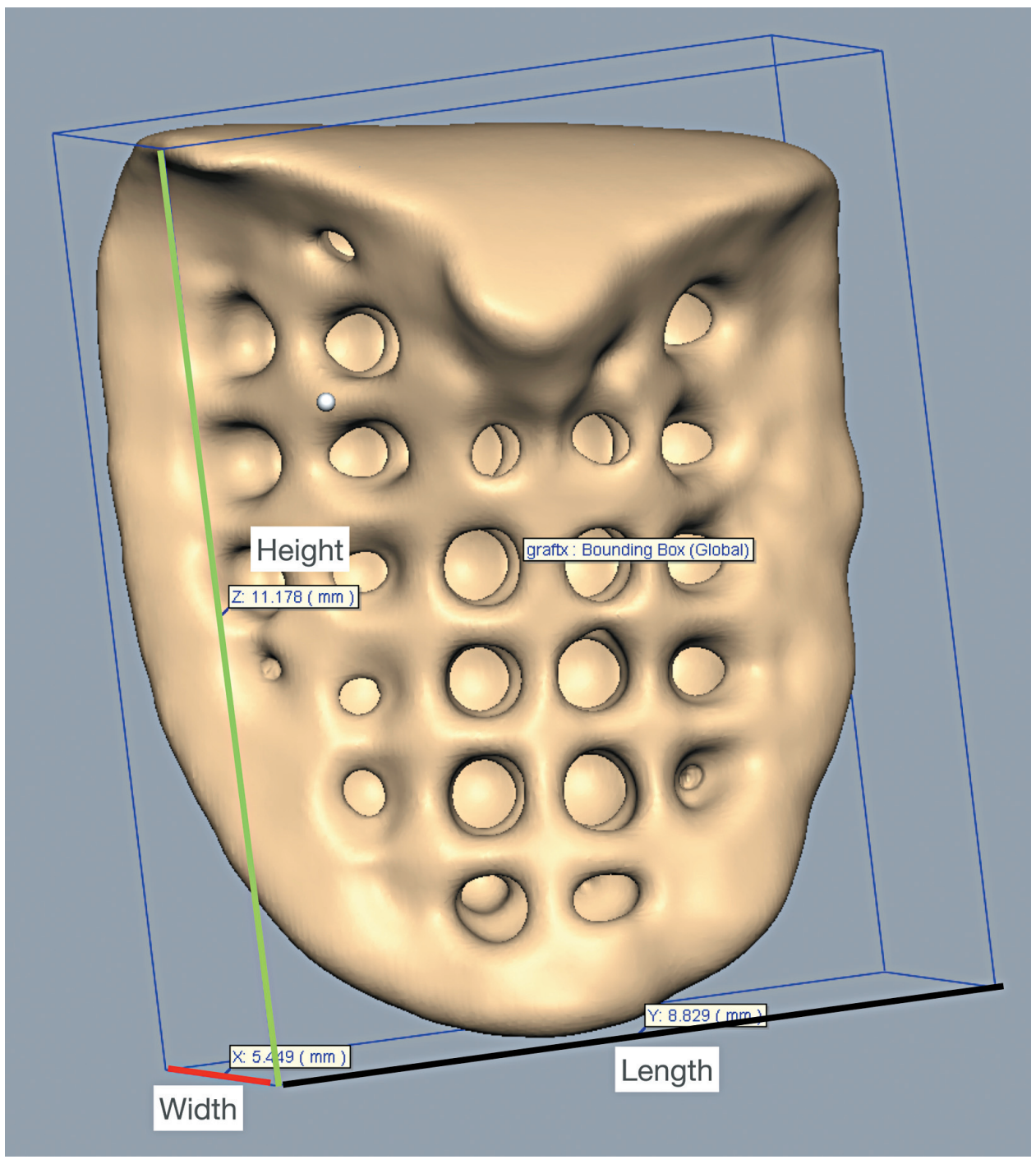

Supplementary Fig. S1 Geometric measurement of 3DHA graft in width, length and height using Geomagic freeform Software (3D Systems, USA). Data was presented as millimeter (mm).

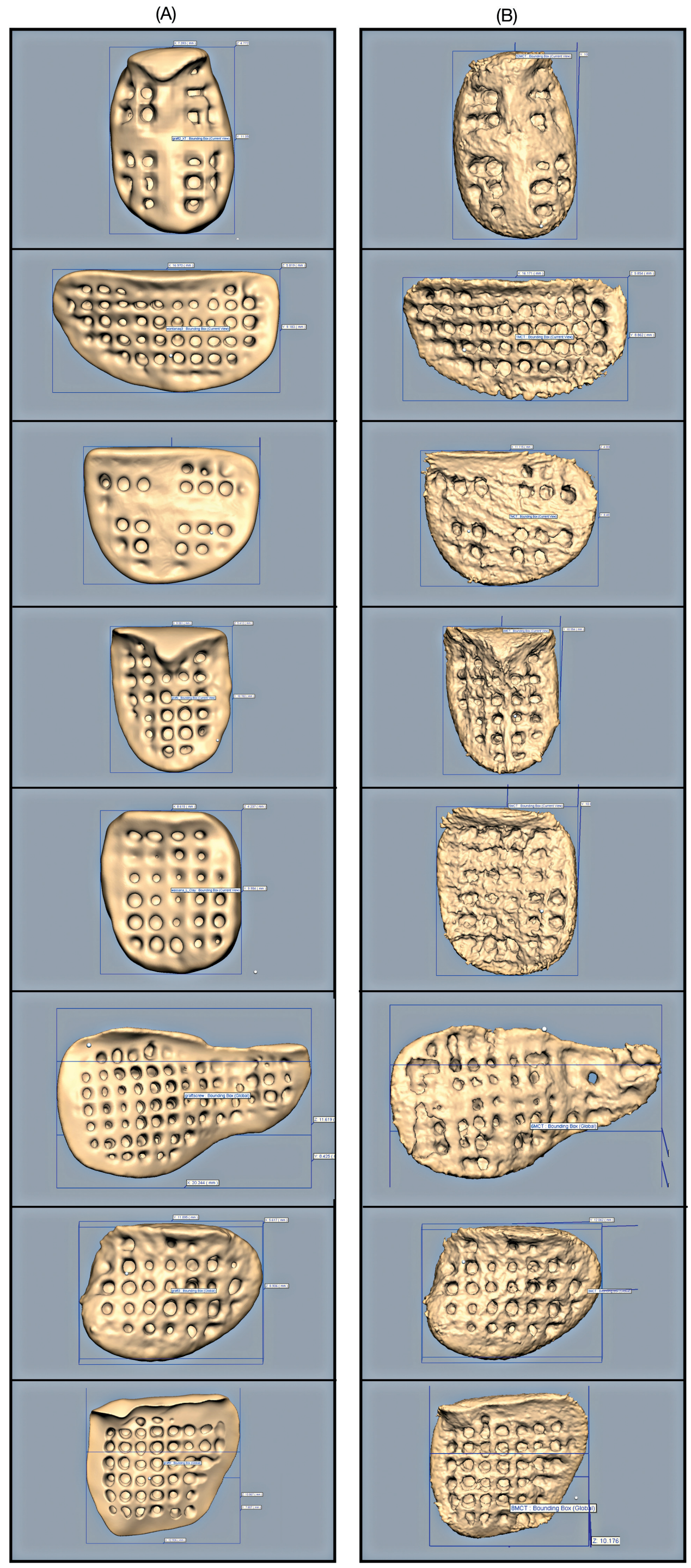

Supplementary Fig. S2 STL files of 3DHA virtual model (**A**) and scanned μCT (**B**). Each figure represents 3DHA virtual model scanned μCT for each defect (defects 1 to 8).

 © 2025. The Author(s).

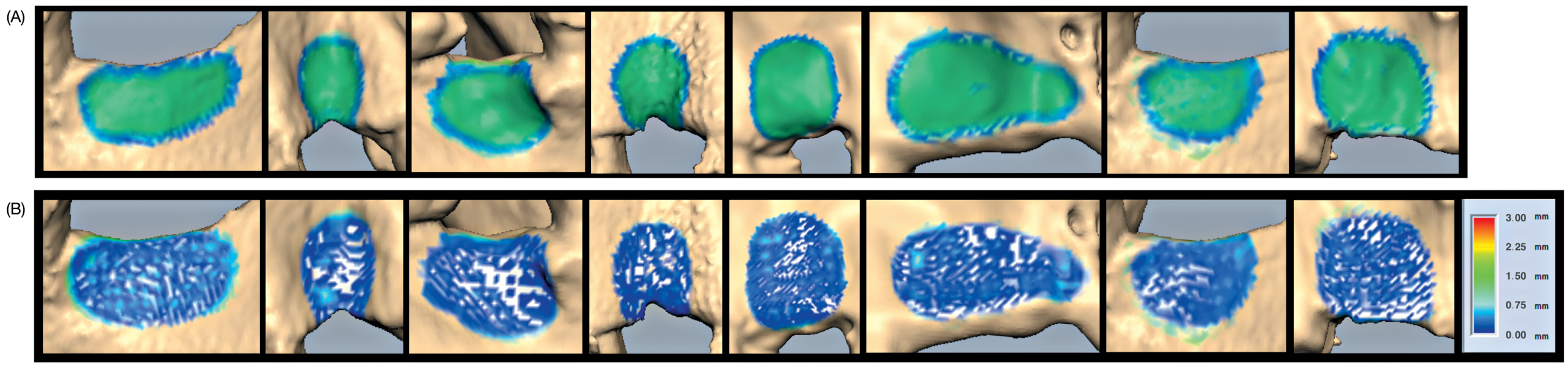

Supplementary Fig. S3 (**A**) The marginal interest area was measured at the distance of 1.25 mm from the marginal edge (blue area). The internal area was measured at the internal graft area at inside 1.25 mm from the marginal area (green area). (**B**) 3D Color maps show gap distances between 3DHA and the defect sites.

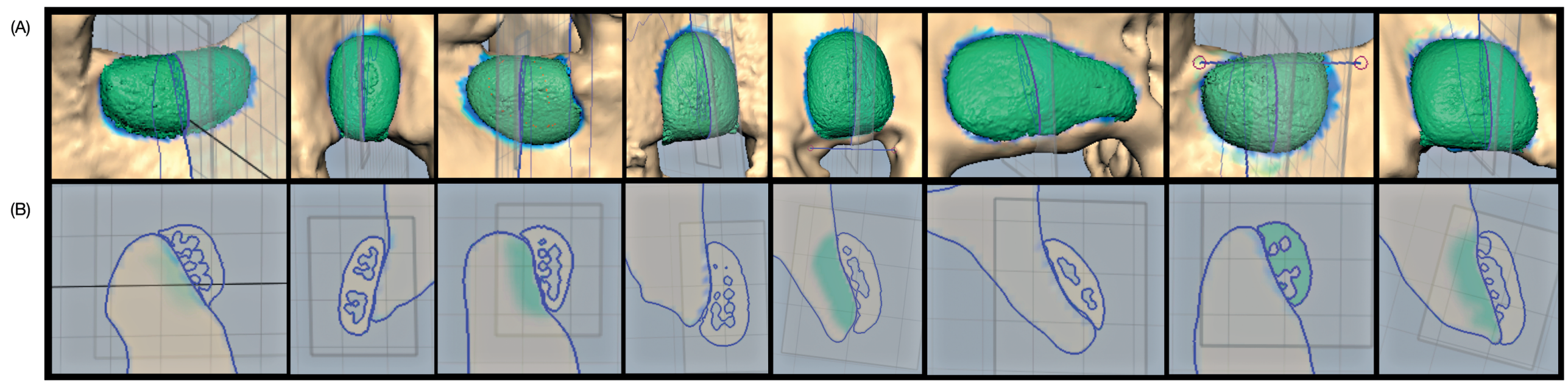

Supplementary Fig. S4 (**A**) Cross sectional plane at middle part of 3DHA. (**B**) Cross sectional views show the fitness of 3DHA and the defect sites.

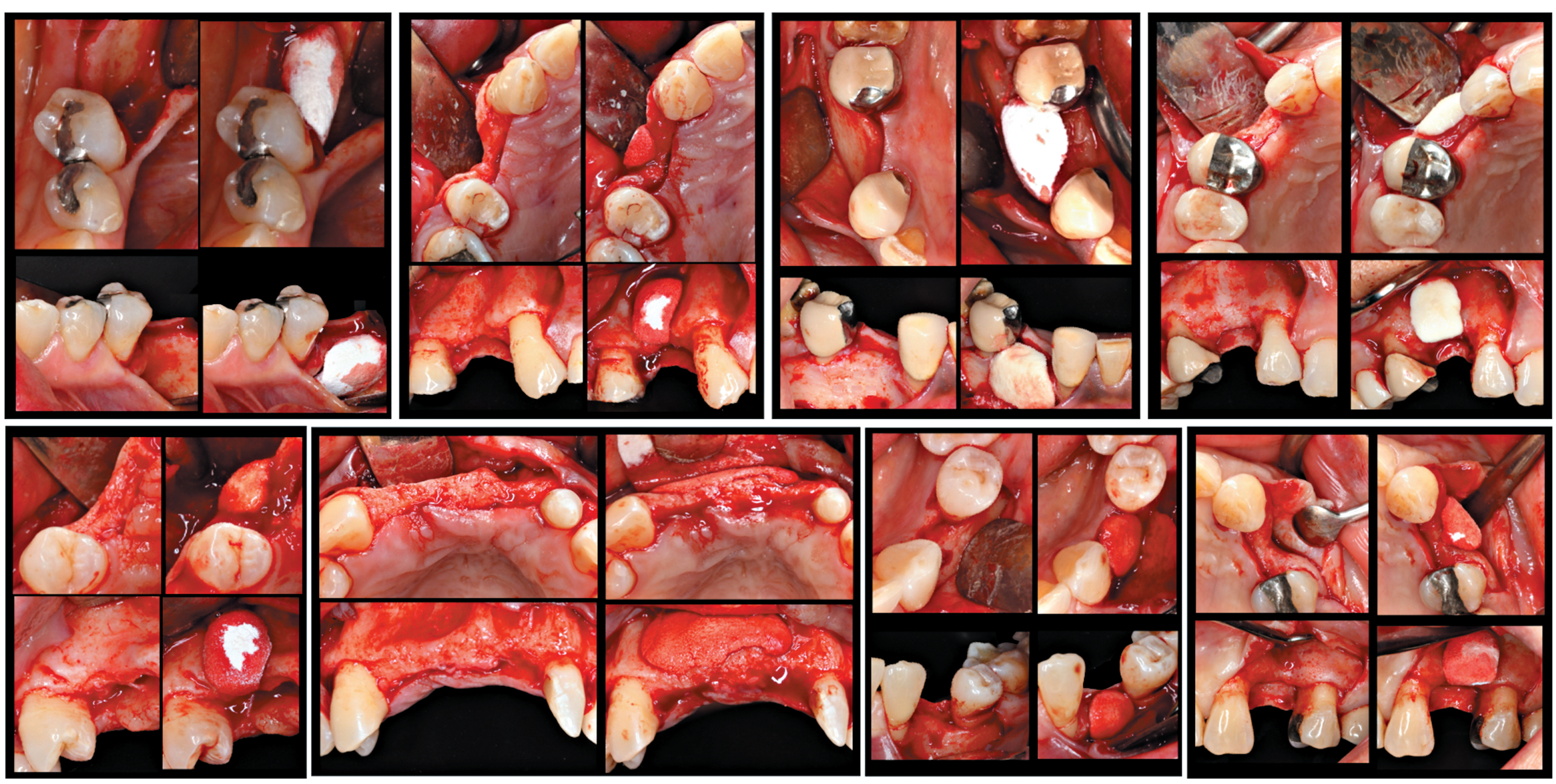

Supplementary Fig. S5 Clinical appearances of grafted areas showed the 3DHA were fitted to the defects.

 © 2025. The Author(s).

Supplementary Table S1 Descriptive statistics of root mean square value (mm) presented by mean ± SD for overall, margin, inner, and contour areas of the 3DHA graft

	Mean ± SD	95% CI		ICC
		Lower bound	Upper bound	
Overall	0.19 ± 0.04	0.16	0.23	0.99
Margin	–0.22 ± 0.25	–0.25	–0.20	0.93
Inner	–0.08 ± 0.18	–0.10	–0.06	0.87
Contour	0.03 ± 0.11	0.02	0.04	0.89

Abbreviations: 3DHA, three-dimensional-printed hydroxyapatite; CI, confidence interval; ICC, intraclass correlation coefficient; SD, standard deviation.

Supplementary Table S2 Repeated-measures ANOVA (multiple comparisons) test of overall, between margin and inner region

	Mean difference (mm)	95% CI of difference	*p*-Value
Overall vs. Margin	0.4174	0.2232 to 0.5982	< 0.0001
Overall vs. Inner	0.278	0.1942 to 0.4123	< 0.0001
Overall vs. Contour	0.1639	0.0838 to 0.3025	< 0.0001
Margin vs. Inner	–0.1393	–0.1703 to –0.0641	< 0.0001
Margin vs. Contour	–0.2534	–0.2903 to –0.1960	< 0.0001
Inner vs. Contour	–0.1141	–0.1774 to –0.0835	< 0.0001

Abbreviations: ANOVA, analysis of variance; CI, confidence interval.

Supplementary Table S3 Gap distances between 3DHA and defect

Defect	Mean Gap distances (mm; mean ± SD)	
	Marginal area	Internal area
1	0.12 ± 0.63	0.35 ± 0.46
2	0.73 ± 0.32	0.44 ± 0.47
3	0.08 ± 0.67	0.57 ± 0.53
4	0.18 ± 0.31	0.44 ± 0.25
5	0.12 ± 0.42	0.36 ± 0.34
6	0.08 ± 0.46	0.40 ± 0.37
7	0.07 ± 0.23	0.19 ± 0.14
8	0.13 ± 0.54	0.24 ± 0.73
Mean ± SD	0.19 ± 0.22	0.37 ± 0.12

Abbreviations: 3DHA, three-dimensional-printed hydroxyapatite; SD, standard deviation.

 © 2025. The Author(s).
